# Healthy Aging Requires a Healthy Home Care Workforce: the Occupational Safety and Health of Home Care Aides

**DOI:** 10.1007/s40572-021-00315-7

**Published:** 2021-05-12

**Authors:** M. M. Quinn, P. K. Markkanen, C. J. Galligan, S. R. Sama, J. E. Lindberg, M. F. Edwards

**Affiliations:** Safe Home Care Project, Lowell Center for Sustainable Production and Department of Public Health, University of Massachusetts Lowell, 600 Suffolk Street, Suite 520, Lowell, MA 01854 USA

**Keywords:** Home health care, Home care, Home health aides, Home care aides, Occupational safety and health, Patient safety

## Abstract

**Purpose of Review:**

To identify important home care (HC) aide occupational safety and health (OSH) hazards and examine how addressing these can improve aide health and the delivery of HC services overall. Specifically, this review seeks to answer: Why is HC aide OSH important? What are the most significant OSH challenges? How can improving HC aide OSH also improve the safety and health of their clients? What implications do the findings have for future research?

**Recent Findings:**

HC is one of the fastest growing US industries. Aides comprise its largest workforce and are increasingly needed to care for the rapidly aging population. There is an aide shortage due in part to instabilities in HC work organization and to serious job-specific hazards, resulting in aides losing work time.

Recent social, economic, and technological factors are rapidly changing the nature of HC work, creating OSH hazards similar to those found in nursing homes. At the same time, aides are experiencing social and economic inequities that increase their vulnerability to OSH hazards. These hazards are also a burden on employers who are challenged to recruit, retain, and train aides. OSH injuries and illness interrupt the continuity of care delivery to clients. Many OSH hazards also put HC clients and families at risk.

**Summary:**

A new framework and methodologies are needed to assess aide and client safety together in order to guide future HC research, policies, and practices. Government, industry, and labor commitment is needed to fund and coordinate a comprehensive, multidisciplinary research program.

## Introduction

Home care (HC) workers provide essential health and personal care supportive services that enable people to live at home rather than receive care in a nursing home or other facility. The great majority of those who receive HC are people over age 65 years, as well as those of all ages with illness or disabilities. The importance of home-based care as an alternative to facility-based care was underscored when the COVID-19 pandemic began: elders were at high risk of severe COVID-19 infection [1], and nursing homes had high infection rates [2–5]. At the same time, most of the HC workforce is composed of aides, who also are vulnerable to COVID-19 due to their work exposures and to their experience of social and economic inequities (housing where social distancing is not possible, limited health care access) that present infection risks [6, 7, 8••, 9]. Many elders were afraid to have aides enter their homes, and many aides were concerned about caring for those with COVID-19 in the home-work environment where infection prevention measures were not well established [10••]. Overall, the pandemic made it clear that a healthy and safe workforce is needed to ensure healthy and safe care to millions of people in their homes.

This paper reviews key scientific literature and policy data to determine: Why is HC aide occupational safety and health (OSH) important? What are the major HC aide OSH hazards and preventive interventions? How do aide OSH hazards impact the safety of HC clients and their families? How can accounting for aide and client safety together improve HC services overall?

## The Structure of Home Care Work

In the USA, there are numerous occupational titles for aides employed in HC, including jobs reimbursed through medical insurance and usually designated as home healthcare, such as home health aide, certified nursing assistant and hospice aide, as well as jobs reimbursed through social services including personal care aide, personal care attendant, and homemaker. For brevity in this paper, the term “HC aide” refers to the full range of aide occupational titles. While there are differences in job tasks among occupational titles, usually related to the degree of medical support provided, there is also considerable overlap with respect to tasks that present OSH risks. Most aides assist individuals in their home with Activities of Daily Living such as bathing, dressing, eating, caring for incontinence, and transferring to and from a bed or chair [11]; they also assist with Instrumental Activities of Daily Living including household work such as cleaning and disinfecting home environmental surfaces, laundry, and food preparation [12••]. Aides may be employed by a business called an agency or hired directly by the care recipients or their family, which is called a consumer-directed program. In the USA, HC recipients are called patients, clients, or consumers; for brevity, “client” refers to all HC recipients.

The wages of most HC aides, whether employed via agencies or directly by clients, come from the Centers for Medicare and Medicaid Services reimbursements and to a lesser degree from healthcare insurance [13] or clients who pay directly (private pay). The Centers for Medicare and Medicaid Services reimbursement schedule is negotiated in advance, and employers faced with sudden changes in demands, like new COVID-19 safety training, often find themselves absorbing unexpected costs [14].

## Social and Medical Determinants of Home Care Work

The confluence of several social, economic, and technological forces is driving the urgent need for HC [13]. Populations in the USA and globally are aging rapidly, and there are comparatively fewer people in younger age groups [15, 16]. From 2010 to 2019, the USA experienced a 34% increase in the 65-and-older population, while those 15–64 years, the working population and age group most likely to be caregivers, increased by only 3.1% [17]. Nearly 17 million Americans living in the community require assistance completing self-care and other daily tasks due to physical, cognitive, developmental, and/or behavioral conditions [12••], and 70% of adults over age 65 will need paid long-term support services at some time before they die [18].

Technological advances are enabling sophisticated procedures at home [19, 20]. At the same time, people are living longer with complex healthcare needs (80% of Americans over 65 years have one or more chronic conditions [21]). Aides do not perform medical procedures directly; however, they interact with medical equipment as they perform care and cleaning tasks. Even if an aide provides care for an older client recovering from hip replacement surgery, that client may also have diabetes, asthma, or cancer requiring medical technologies.

Social and economic forces also are driving the delivery of medical and supportive care into the home [20, 22]. The majority of Americans prefer care at home, and clinicians and insurers are recognizing the medical and financial advantages [23, 24]. This preference for HC extends through end-of-life. Data from 2003 to 2017 show that Americans now prefer to die at home; as of 2016, more Americans died at home than in hospitals, nursing homes, or any other facility [25]. Home has surpassed the hospital as the most common place of death in the USA for the first time since the early 20th century [26].

At the same time the demand for HC is increasing, the supply of traditional home-based caregivers, women aged 15 to 64, is decreasing because most women are now in the paid labor force—67% of US women 15–64 years, 52% globally [17]. This caregiver gap is occurring worldwide and is bringing to light the often invisible care work that women have always done [27, 28].

## Home Care Aide Demographics, Income, and Conditions of Work

There are 3.44 million HC aides in the USA, the great majority are women (87%), and increasingly people of color (62%) and immigrants (31%) [29]. The need for HC aides is creating a global migration from lower to higher income countries [28]. Despite their social importance, however, HC aide jobs are among the lowest paid, with a median annual income of $25,280 in 2019 [30]. At that wage, a full-time HC aide trying to support a family of four would be living below the poverty line ($26,500) [31].

## History of Home Care Aide Income and Structural Inequities

The low wages and social position of HC aides cannot be understood without examining the social and economic inequities within home-based services resulting from systemic gender and racial discrimination. Care work, the work of women in nearly every culture, has often been viewed as unproductive, less valuable, and not warranting wages competitive with work traditionally performed by men [27, 32]. Race too has played a critical role in how HC work is valued. Low HC aide wages were structured into the first federal occupational classifications developed by the Roosevelt administration’s Department of Labor in 1938. The Fair Labor Standards Act (FLSA), a cornerstone of the “New Deal,” was designed to protect workers through a minimum wage and other benefits. Legislators from southern states, with economies first built on slavery and later sustained by Jim Crow era laws, refused to support a law that would require Blacks doing the same job as Whites to earn the same wages [33]. However, a law explicitly discriminating on the basis of race is unconstitutional under the 14^th^ amendment, ratified in 1868 and providing all citizens with “equal protection under the laws.” In order to pass the FLSA, legislators compromised to create occupational categories that were exempted from protections. Among these were agricultural workers, the main occupation of Black men, and domestic service workers, the main occupation of Black women. Thus, the FLSA codified “race-neutral” language in a concession to southern legislators, who were needed to pass New Deal legislation [33]. In 1974, Congress revised the FLSA to expand coverage to include domestic service workers. One year later, however, the US Department of Labor “interpreted [this] new amendment to exempt home-care workers, even employees of for-profit entities, by misclassifying them as elder companions, akin to babysitters” [34, 35]. This amendment became known as the “companionship rule” [36]. Thus, the companionship classification of domestic service workers, including HC occupations, carried forward the legacy of the Antebellum South [33]. The companionship rule for HC occupations remained in place for more than 75 years, until the US Supreme Court overturned it in 2015, finally requiring HC aides to be paid the federal minimum wage.

Although no longer sanctioned by federal law, HC aide work continues to be viewed by many as akin to babysitting with significant consequences for aides’ conditions of work. Like babysitters, aides are frequently hired part-time on an hourly basis with inconsistent hours [12••, 29, 37]. This work organization creates instability in HC services delivery and is a burden on HC clients and employers, as well as aides. For example, clients have reported problems with the quality of their care arising from aides’ work conditions and work organization [38]. Many HC employers are struggling to recruit and retain adequate numbers of HC aides [12••, 39]. An unstable workforce increases business costs and management complexities, including for job training and the provision of OSH. Aides frequently reported that they would like to stay in HC, but low wages, irregular and uncertain hours, and lack of employer-provided benefits made it difficult [29], thus contributing to high aide workforce turnover [14]. Industry reports have also concluded that the poor quality of HC aide jobs contributes to the workforce shortage [12••, 39].

Economic and work organizational factors present health and safety risks in themselves, and they intensify the risks of harm from specific job hazards. They also are structural obstacles to professionalizing the workforce even as the needs for more workers with more training and ability to deliver more specialized services are urgent. Understanding the historical roots of poor pay and unstable working conditions should inform the changes needed to recruit, retain, and professionalize the HC aide workforce.

## Organization of the Home Care Workplace and Implications for Occupational Safety and Health Regulation

Home care work differs from that in hospitals, nursing homes, and other healthcare facilities because the environment where care services are delivered is a private home. In facilities, healthy and safe working conditions are regulated by the US Department of Labor Occupational Safety and Health Administration (OSHA). Home care agencies are required to comply with OSHA regulations for aspects of HC work outside the home, but when the home becomes a workplace, there are significant limitations on agency employers’ and OSHA’s ability to ensure HC worker OSH. These limitations can be evaluated using the central OSH hazard prevention model called the “hierarchy of controls,” a scale with the top representing the most effective controls, which eliminate hazards from the work environment, ranging to the least effective, controlling hazards at the individual level (Table 1). Applying the model to HC, it is seen that agencies’ and OSHA’s actions to improve OSH for aides are often limited to the least effective interventions.

**Table 1:**
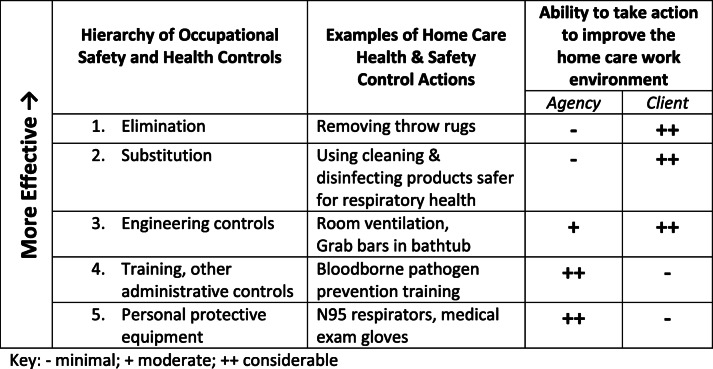
Hierarchy of the effectiveness of occupational safety and health control actions applied to the home care work environment

Aide OSH training, although not at the top of the hierarchy of controls, is essential and can reduce risks [40–42, 43••]; however, even with adequate training, aides are limited in controlling their work environment in a private home [44]. For example, one study [45] found that while HC agencies complied with requirements for training and exposure control plans under the OSHA Bloodborne Pathogens Standard, they were not able to ensure other requirements of the standard for the use of sharps with injury prevention features [46], nor could they guarantee that sharps were properly stored and disposed in the home by clients [45, 47, 48]. There is a delicate balance between protecting client autonomy and creating safer working and care conditions. Advocates for elders and people with disabilities have sometimes viewed HC aide safety, particularly interventions which alter the home-work environment, as conflicting with clients’ rights and autonomy [49]. Instead, an approach is needed to align aide and client safety and well-being and find comprehensive solutions that work for both.

## Home Care Job Rewards

Aides have reported higher levels of job satisfaction, especially compared to work in a nursing home or other facility [50, 51]. Foremost was their ability to develop rewarding relationships with clients and their families, thereby creating a sense of meaning and significance. Additional job rewards were the ability to work autonomously and to have flexible work schedules [50, 51]. Social support from agency and case managers and union staff can also positively impact aide jobs [52–54].

## Home Care Job Hazards

The home-work environment presents OSH risks rarely found in facilities, including spaces too small or cluttered to perform care work safely, aggressive unrestrained pets, bedbugs and other pests, loaded firearms, needles used by the client or other household members and discarded around the home, tobacco smoking, clients smoking while on oxygen, slippery or unsafe surfaces indoors and outdoors, and transportation accidents during work travel [47, 50, 51, 55••, 56, 57••].

## Home Care Injuries and Work-Related Musculoskeletal Disorders

While the home-work *environment* differs in important ways from that in healthcare facilities, work *tasks* that involve close interactions, such as patient/client handling, are similar to those performed by aides in nursing homes, and many of the injury risks are the same. It is challenging to get complete national injury statistics for HC, especially from the consumer direct-hire programs, because reporting systems are under-developed [55••]. Washington state offers a valuable data source because it has a single, comprehensive workers’ compensation system that covers most of its industries, including all types of paid HC. A limitation is that workers’ compensation data represent only well-recognized, very serious injuries; occupational illnesses are seldom compensated [52]. Data from Washington state workers’ compensation claims, 2012–2016, showed that the overall injury rate in home health services was 259/10,000 Full-Time Equivalent Employees (FTE), slightly higher than in nursing homes during the same period, 241/10,000 FTE [58••]. There were 7179 HC workers’ compensation claims, totaling $86 million [58••]. The patterns of compensable injuries did not vary importantly among different types of HC services (home health services, personal care services provided by agency-hired aides or personal care services provided by consumer-hired aides). The authors concluded that all HC providers likely experienced similar injury risk factors. The great majority of injuries resulting in workers’ compensation claims were work-related musculoskeletal disorders (WMSD), followed by falls on the same level and injuries due to violence. Struck-by injuries and transportation injuries also resulted in high costs and lost work time [58••]. Assessments of HC aide fall injuries pointed to preventive interventions including reducing slippery surfaces and providing aides with slip-resistant shoes [59].

Back injuries occurred most frequently, according to the Washington state workers’ compensation data, followed by shoulder and neck injuries. These findings are similar to other HC aide studies [51, 60–64]. A survey of nearly 3500 aide visits to clients’ homes found that many lacked lifting or transfer devices or other assistive equipment when needed for client care [51]. National Institute for Occupational Safety and Health (NIOSH) guidelines specify that healthcare workers should not lift more than 35 pounds, under optimum ergonomic conditions [65, 66]. Occupational ergonomics research has established that back injury prevention programs based only on body mechanics and “proper lifting techniques” do not reduce WMSD [66]. Instead, comprehensive Safe Patient Handling and Mobility (SPHM) programs are needed in HC, including development of policies and procedures and successful implementation of low-tech and powered-tech assistive equipment [67]. Many hospitals and nursing homes are implementing “zero-lift” policies that rely on assistive technologies as part of their SPHM programs, but the equipment used in facilities frequently is not feasible in HC, and innovation in assistive technology design is needed [64].

## Violence

Home care aides’ experiences of work-related violence were so severe and frequent as to rank third among all Washington state workers’ compensation claims for in-home services, 2012–2016 [58••]. Furthermore, the number of violence claims doubled since the previous 4-year period. A study of 934 aides’ experiences of verbal abuse from clients and family members identified risk factors: homes with too little space for the aide to work, caring for clients with dementia, caring for clients with limited mobility, and having a client with an unclear plan for care delivery [68••]. Aides reporting verbal abuse were 11 times as likely to also report physical abuse [68••]. Aides who reported often being asked to do tasks outside their job duties were more likely to report both verbal and physical abuse and pain/injury with lost work time or medical care. These aides were also less likely to want to remain in their job or to recommend it to others [69]. A potential protective intervention was identified: aides having predictable work hours were less likely to report verbal abuse [68••]. These findings suggest specific changes in work organization and training to reduce violence [70].

In a literature review of violence interventions for home healthcare workers, safety and health training was important in increasing workers’ confidence and knowledge about violence prevention [71]. Other studies have reported similar findings on the importance of violence prevention training in HC [41, 72]. Comprehensive violence prevention programs have been developed for hospitals and other facilities, and these should be adapted for HC work [72–77].

## Sharps Injuries and Bloodborne Pathogen Exposures

Many procedures performed at home use sharp medical devices such as needles, syringes, and lancets, collectively called “sharps.” Needlesticks and other injuries with sharps previously used by clients or others in the household present a risk of serious bloodborne pathogen exposures to HC workers, including Hepatitis B and C and HIV [78••]. While aides typically do not use sharps, they perform care tasks that present risks of being stuck or cut with a used sharp if not stored and disposed properly. Although outside their job duties, aides have reported being asked by clients or their families to perform medical procedures using sharps, for example to administer vitamin or insulin injections [50, 51]. A meta-analysis of HC aide sharps injuries estimated that HC aides have a 2% annual risk of experiencing at least one sharps injury [78••]. Risk factors included helping a client use a sharp, observing used sharps lying around the home, and caring for physically aggressive clients. Aides hired directly by clients, male aides, and aides who were immigrants had higher risks than their counterparts [79]. Not only are HC workers at risk, but so too are clients, their family members, or visitors within the home, as well as waste disposal workers. Risks of sharps injuries are also costly for HC agency employers because they require resources for training, management, reporting, prophylactic treatment, lost work time, and workers’ compensation insurance. Ideally, preventive interventions should be aimed at eliminating sharps use, including through the development of needleless medical devices and procedures. In the meantime, sharps with injury prevention features should be used in HC [45].

## Infection Hazards

Quantitative assessments of infectious exposures and diseases related to HC are limited. One study conducted a microbiology assessment of home environmental surfaces to evaluate the presence of two pathogens frequently associated with healthcare acquired infections (HAI), *Staphylococcus aureus* (*SA*), and *Clostridium difficile* (*C. diff*). Among 46 homes in elder housing, 30% of which were receiving HC services, *C. diff* was identified in only 1 home, and 7 homes were found to have *SA*, only one of these was methicillin resistant (MRSA). The authors concluded pathogen exposures were relatively low in this elder housing compared to households with people of all ages [80].

A few studies have estimated the prevalences of infectious exposures occurring in clients’ homes and found the most common to be body fluids (saliva, respiratory mucus, sweat, and feces) and pet waste [51, 81]. In addition to infection, HC aides must also contend with health hazards from chemicals in cleaners that they use at nearly every client visit (80% of visits involve cleaning, according to a survey [51]). Cleaning products in common use by HC aides and clients contain respiratory irritants and sensitizers including bleach and quaternary ammonium compounds [55••, 82].

Home care aides have often been overlooked as part of the medical team [83]; however, their role in healthcare gained new visibility during the COVID-19 pandemic when hospitals were over capacity, and there was concern about increased risk of infection in congregate care settings [3–5, 23, 84]. A survey on COVID-19 impacts in HC found that agencies began providing services to clients with COVID-19 or under investigation for COVID-19 since the outset of the pandemic in March 2020 [10••]. Aides too were diagnosed with COVID-19 or quarantined because they were in contact with a case in the community or during their HC work. The survey also found that 54% of aides working for HC agencies also worked in nursing homes or congregate care settings, presenting a second source of occupational exposure [10••].

At the same time, households have been identified as high-risk settings for the transmission of severe acute respiratory syndrome coronavirus 2 (SARS-CoV-2) [85]. While no data are available on transmission of SARS-COV-2 specifically to HC aides by clients, or vice versa, a few studies found that the secondary attack rate for SARS-CoV-2 among members of the same household is substantially higher than among other contacts such as healthcare workers, workplace contacts, and non-household contacts [86]. A recent study measured SARS-CoV-2 in air samples from households and inpatient hospital rooms, both selected based on the presence of SARS-CoV-2 positive patients. Household samples were eight times more likely to test positive for the virus than inpatient samples. Room ventilation (air changes per hour) was the main difference between these settings [86]. Further research is needed to understand how to safely care for COVID-19 patients at home.

Clearly, COVID-19 vaccination of HC aides is a high priority. It is concerning therefore that aides have had low rates of vaccination against seasonal flu [8••]. Along with medical considerations, effective vaccination programs need to address the economic and social determinants of HC aide infection risks, their access to vaccinations, and their concerns about accepting vaccinations [8••, 87].

## Occupational Safety and Health Training

The importance of OSH training for HC aides is underscored in nearly every study, including those providing qualitative data in the words of aides and clients [38, 50, 52, 88••]. Despite not being at the top of the hierarchy of health and safety controls (Table 1), it is clear that, given aides’ independent work in highly variable home-work environments, training is an important intervention. A recent comprehensive training program model, developed using a NIOSH Total Worker Health approach, combined OSH with health promotion [42, 43••]. The program’s effectiveness was evaluated using a randomized trial design and found significant aide improvements in the use of ergonomic tools and techniques, safety communication with clients, hazard corrections in homes, and improved diet and physical strength. Additionally, clients reported that aides’ safety behavior improved [42, 43••]. These results suggest that health and safety training builds important skills contributing to professionalizing HC aide jobs.

## Conclusions and Recommendations

Major social, economic, and technological trends are driving the need for HC and its largest workforce, HC aides. There is already a HC aide shortage, and the industry faces challenges to recruit and retain them, mainly due to instabilities in the organization of HC work including OSH hazards. Many HC aide OSH hazards also put clients at risk, and aide injuries and illness disrupt care services. This review has identified several main conclusions with implications for future research.

1. *HC aides experience serious OSH hazards, injuries, and illnesses that are a burden for them as well as for employers and clients.* These include work-related musculoskeletal disorders; injuries from falls; physical and psychosocial injuries from violence at work; risks of bloodborne, respiratory, and other infections; and harmful indoor air quality related to secondhand smoke, disinfectants, and other chemicals. Many OSH hazards derive from and are intensified by social and economic inequities experienced by HC aides. OSH injuries and illnesses also are costly for employers and contribute to the poor conditions of HC aide work that make it difficult for employers to recruit and retain a workforce. Many OSH hazards may also harm clients, for example when there are client injury risks related to handling and mobilization, sharps injuries to householders, COVID-19 and other infection risks from inadequate infection prevention in the home, unhealthy home environmental air quality, and home safety hazards related to falls and fires. High-quality HC services depend on the abilities of aides and clients to develop trusting relationships; when an aide loses work time, clients and families suffer from the discontinuity of care. Intervention research is needed to evaluate new approaches to engaging HC aides and clients in HC safety and health.
2. *The rewarding aspects of HC aide work present opportunities for enhancing job satisfaction and making jobs more attractive for improved workforce recruitment and retention.* Research is needed to evaluate more fully the positive aspects of HC aide work so that they can be used as a starting point for improvements in job design and work innovations.
3. *A framework and process to engage aides, clients, and families to account for their joint safety and health is needed to guide HC safety research and policy.* In addition to providing more comprehensive and durable safety solutions, engaging clients and their families would improve the ability to make safety changes in the home that are higher on the hierarchy of OSH controls and therefore more effective overall.
4. *Home care aides should be considered members of the broader healthcare team*. HC aides can make important contributions to conversations about home safety and to care plans for their clients, but aides seldom have a direct line of communication with the primary care physician, physical therapist, or others on the care team [10••, 89]. At the same time, connecting HC with healthcare should be achieved while respecting the preference of the disability community for a social, independent living model rather than a medical model which risks defining clients as an illness. Because of the frequent and close contact, aides often are the first to recognize a change in clients’ health or cognitive status. Conversely, clients may have new illnesses or medical technologies about which the aide is given no information. This highlights opportunities for initiatives to upskill and professionalize aide jobs. Safety and health skill building can be mutually beneficial for improving client health and offering expanded career pathways for aides.
5. *New, comprehensive programs of HC safety and health research are needed.* The existing literature on HC OSH is limited, especially given the social importance of the work, size of the workforce, and the variability and rapid growth of the industry. Quantitative measurements of hazards in the home-work environment are lacking as are data sets of large populations and international comparative studies of HC delivery systems. The last US national survey [90] was conducted in 2007; comprehensive quantitative and qualitative data on HC OSH and employment conditions are needed. These new studies should simultaneously assess the health, safety, and well-being of clients and aides. Research initiatives that address both HC aides’ and clients’ needs will require coordination among government funding agencies and foundations. Current research funding is too limited and narrowly focused to make substantial HC safety and health improvements for all stakeholders.

The COVID-19 pandemic presents an opportunity to reimagine care work [91]. The World Health Assembly has designated 2021 as the International Year of Health and Care Workers [92]. This is the time to transform HC health and safety.

## Data Availability

Not applicable.

## References

[1] Hao B, Sotudian S, Wang T, Xu T, Hu Y, Gaitanidis A, et al. Early prediction of level-of-care requirements in patients with COVID-19. Elife. 2020;9. 10.7554/eLife.60519. 10.7554/eLife.60519 PMC7595731 33044170

[2] Bagchi S, Mak J, Li Q, Sheriff E, Mungai E, Anttila A (2021). Rates of COVID-19 among residents and staff members in nursing homes—United States, May 25-November 22, 2020. MMWR Morb Mortal Wkly Rep.

[3] Dora AV, Winnett A, Jatt LP, Davar K, Watanabe M, Sohn L (2020). Universal and serial laboratory testing for SARS-CoV-2 at a long-term care skilled nursing facility for veterans—Los Angeles, California, 2020. MMWR Morb Mortal Wkly Rep.

[4] Kimball A, Hatfield KM, Arons M, James A, Taylor J, Spicer K (2020). Asymptomatic and presymptomatic SARS-CoV-2 Infections in residents of a long-term care skilled nursing facility—King County, Washington, March 2020. MMWR Morb Mortal Wkly Rep.

[5] Roxby AC, Greninger AL, Hatfield KM, Lynch JB, Dellit TH, James A (2020). Detection of SARS-CoV-2 among residents and staff members of an independent and assisted living community for older adults—Seattle, Washington, 2020. MMWR Morb Mortal Wkly Rep.

[6] Abrams EM, Szefler SJ (2020). COVID-19 and the impact of social determinants of health. Lancet Respir Med.

[7] Adams V, Song J, Shang J, McDonald M, Dowding D, Ojo M, et al. Infection prevention and control practices in the home environment: examining enablers and barriers to adherence among home health care nurses. Am J Infect Control. 2020. 10.1016/j.ajic.2020.10.021. 10.1016/j.ajic.2020.10.021 PMC8093314 33157183

[8.••] Silver S, Boiano J, Li J (2020). Patient care aides: differences in healthcare coverage, health-related behaviors, and health outcomes in a low-wage workforce by healthcare setting. Am J Ind Med.

[9] Allison TA, Oh A, Harrison KL (2020). Extreme vulnerability of home care workers during the COVID-19 pandemic—a call to action. JAMA Intern Med.

[10.••] Sama SR, Quinn MM, Galligan CJ, Karlsson ND, Gore RJ, Kriebel D, et al. Impacts of the COVID-19 pandemic on home health and home care agency managers, clients, and aides: a cross-sectional survey, March to June, 2020. Home Health Care Manag Pract. 2020:1084822320980415. 10.1177/1084822320980415**Documents early pandemic home care aide COVID-19 exposures.**

[11] U.S. Department of Health and Human Services. Long term care pathfinder. What is long term care and who needs it? 2021. https://acl.gov/ltc/basic-needs/pathfinder. Accessed 30 Mar 2021.

[12.••] Paraprofessional Healthcare Institute. Caring for the future: the power and potential of America's direct care workforce. 2021. http://phinational.org/resource/caring-for-the-future-the-power-and-potential-of-americas-direct-care-workforce/. Accessed 30 March 2021. **Excellent source of national statistics on the direct care workforce, including home care aides, and on long term care needs of the U.S. population.**

[13] Paraprofessional Healthcare Institute. U.S. Home Care Workers: key facts 2019. https://phinational.org/resource/u-s-home-care-workers-key-facts-2019/. Accessed 30 March 2021.

[14] Gleason H. Setting the agenda: data driven advocacy to address home care aide policy. The Home Care Aide Foundation; 2018. http://commcorp.org/wp-content/uploads/2018/02/Resource_HCAF_Setting-the-agenda-data-driven-advocacy-to-address-home-care-aide-policy_Feb-2018.pdf. Accessed: 30 Mar 2021.

[15] The World Bank Group. Population ages 15-64, total. World Bank staff estimates using the World Bank's total population and age/sex distributions of the United Nations Population Division's World Population Prospects: 2019 Revision; 2019. https://data.worldbank.org/indicator/SP.POP.1564.TO. Accessed: 30 Mar 2021.

[16] The United Nations, Department of Economic and Social Affairs: population dynamics. World population prospects 2019. Geneva, Switzerland: The United Nations; 2019. https://population.un.org/wpp/. Accessed: 30 Mar 2021.

[17] Rogers L, Wilder K. Shift in working-age populations relative to older and younger Americans. U.S. Census Bureau; 2020. https://www.census.gov/library/stories/2020/06/working-age-population-not-keeping-pace-with-growth-in-older-americans.html. Accessed: 30 Mar 2021.

[18] Office of the Assistant Secretary for Planning and Evaluation (ASPE), Office of Disability AaL-TCP. ASPE Research Brief. What is the lifetime risk of needing and receiving long-term services and supports. U.S. Department of Health and Human Services; April 2018. https://aspe.hhs.gov/pdf-report/what-lifetime-risk-needing-and-receiving-long-term-services-and-supports. Accessed 30 Mar 2021.

[19] Markkanen P, Galligan C, Quinn M (2017). Safety risks among home infusion nurses and other home health care providers. J Infus Nurs.

[20] Hostetter M, Klein S. Has the time finally come for hospital at home? New York (NY): the Commonwealth fund; 2020. https://www.commonwealthfund.org/publications/2020/jul/has-time-finally-come-hospital-home. Accessed 30 Mar 2021.

[21] Centers for Disease Control and Prevention. Percent of U.S. adults 55 and over with chronic conditions. U.S. Department of Health and Human Services; 2015. https://www.cdc.gov/nchs/health_policy/adult_chronic_conditions.htm. Accessed 30 Mar 2021.

[22] Landers S, Madigan E, Leff B, Rosati RJ, McCann BA, Hornbake R (2016). The future of home health care: a strategic framework for optimizing value. Home Health Care Manag Pract.

[23] Brand S. Care in the home is not one-size-fits-All. The voice of Winter 2020: Home Care Association of America; 2020. p. 7. http://www.associationpublications.com/flipbooks/hcaoa/2020/Winter/6/. Accessed: 30 Mar 2021.

[24] Hess C, Hegewisch A (2019). The future of care work: improving the quality of America's fastest-growing jobs.

[25] Cross SH, Warraich HJ (2019). Changes in the place of death in the United States. N Engl J Med.

[26] Warraich H (2017). Modern death: how medicine changed the end of life.

[27] Duffy M, Armenia A, Stacey CL, Duffy M, Armenia A, Stacey CL (2015). On the clock, off the radar: paid care work in the United States. Caring on the clock: the complexities and contradictions of paid care work.

[28] World Health Organization (2017). Women on the move: migration, care work and health.

[29] Paraprofessional Healthcare Institute. Direct care workers in the United States: key facts. 2020. http://phinational.org/resource/direct-care-workers-in-the-united-states-key-facts/. Accessed 30 Mar 2021.

[30] Bureau of Labor Statistics (2020). Occupational outlook handbook: home health aides and personal care aides.

[31] Office of the Assistant Secretary for Planning and Evaluation, U.S. Department of Health & Human Services. Poverty guidelines 01/15/2021. U.S. federal poverty guidelines used to determine financial eligibility for certain federal programs. 2021. https://aspe.hhs.gov/poverty-guidelines. Accessed 30 March 2021.

[32] Messing K, Neis B, Dumais L. Invisible: issues in women's occupational health: Gynergy Books; 1995.

[33] Perea JF. The echoes of slavery: recognizing the racist origins of the agricultural and domestic worker exclusion from the National Labor Relations Act. 2010. 10.2139/ssrn.1646496. https://papers.ssrn.com/sol3/papers.cfm?abstract_id=1646496. Accessed 30 Mar 2021.

[34] Boris E, Klein J. History shows how 2 million workers lost rights. Time; 2015. https://time.com/3664912/flsa-home-care-history/. Accessed 30 Mar 2021.

[35] Fernández Campbell A. Kamala Harris just introduced a bill to give housekeepers overtime pay and meal breaks. Vox; 2019. https://www.vox.com/2019/7/15/20694610/kamala-harris-domestic-workers-bill-of-rights-act. Accessed 30 Mar 2021.

[36] U.S. Department of Labor, Wage and Hour Division.The Fair Labor Standards Act of 1938, as amended. WH Publication 1318. 2011. https://www.dol.gov/sites/dolgov/files/WHD/legacy/files/FairLaborStandAct.pdf. Accessed 30 Mar 2021.

[37] Osterman P (2017). Who will care for us? Long-term care and the long-term workforce.

[38] Westerberg K, Hjelte J, Josefsson S (2017). Understanding eldercare users' views on quality of care and strategies for dealing with problems in Swedish home help services. Health Soc Care Commun.

[39] Espinoza R. 8 signs the shortage in paid caregivers is getting worse. Paraprofessional Health Insitute; 2017. https://phinational.org/8-signs-the-shortage-in-paid-caregivers-is-getting-worse/. Accessed 30 Mar 2021.

[40] Darragh AR, Lavender S, Polivka B, Sommerich CM, Wills CE, Hittle BA, Chen R, Stredney DL (2016). Gaming simulation as health and safety training for home healthcare workers. Clin Simul Nurs.

[41] Vladutiu CJ, Casteel C, Nocera M, Harrison R, Peek-Asa C (2016). Characteristics of workplace violence prevention training and violent events among home health and hospice care providers. Am J Ind Med.

[42] Olson R, Thompson SV, Elliot DL, Hess JA, Rhoten KL, Parker KN, Wright RR, Wipfli B, Bettencourt KM, Buckmaster A, Marino M (2016). Safety and health support for home care workers: the COMPASS randomized controlled trial. Am J Public Health.

[43.••] Olson R, Hess JA, Parker KN, Thompson SV, Rameshbabu A, Luther Rhoten K, et al. From research-to-practice: an adaptation and dissemination of the COMPASS program for home care workers. Int J Environ Res Public Health. 2018;15(12). 10.3390/ijerph15122777**A model safety and health training program for home care aides based on the NIOSH Total Worker Health approach.** 10.3390/ijerph15122777 PMC6313608 30544530

[44] Wills CE, Polivka BJ, Darragh A, Lavender S, Sommerich C, Stredney D (2016). "Making do" decisions: how home healthcare personnel manage their exposure to home hazards. West J Nurs Res.

[45] Markkanen P, Galligan C, Laramie A, Fisher J, Sama S, Quinn M (2015). Understanding sharps injuries in home healthcare: The Safe Home Care qualitative methods study to identify pathways for injury prevention. BMC Public Health.

[46] U.S. Department of Labor, Occupational Safety and Health Administration. Enforcement procedures for the occupational exposure to bloodborne pathogens. CPL 02-02-0692001. https://www.osha.gov/pls/oshaweb/owadisp.show_document?p_table=DIRECTIVES&p_id=2570. Accessed 30 Mar 2021.

[47] Markkanen P, Quinn M, Galligan C, Chalupka S, Davis L, Laramie A (2007). There's no place like home: a qualitative study of the working conditions of home health care providers. J Occup Environ Med.

[48] Quinn M, Markkanen P, Galligan C, Kriebel D, Chalupka S, Kim H (2009). Sharps injuries and other blood and body fluid exposures among home health care nurses and aides. Am J Public Health.

[49] Institute for Healthcare Improvement. No place like home: advancing the safety of care in the home. Report of an Expert Panel Convened by the Institute for Healthcare Improvement. 2018. http://www.ihi.org/resources/Pages/Publications/No-Place-Like-Home-Advancing-Safety-of-Care-in-the-Home.aspx. Accessed 30 Mar 2021.

[50] Markkanen P, Quinn M, Galligan C, Sama S, Brouillette N, Okyere D (2014). Characterizing the nature of home care work and occupational hazards: a developmental intervention study. Am J Ind Med.

[51] Quinn MM, Markkanen PK, Galligan CJ, Sama SR, Kriebel D, Gore RJ, Brouillette NM, Okyere D, Sun C, Punnett L, Laramie AK, Davis L (2016). Occupational health of home care aides: results of the safe home care survey. Occup Environ Med.

[52] Schoenfisch AL, Lipscomb H, Phillips LE (2017). Safety of union home care aides in Washington State. Am J Ind Med.

[53] Yoon S, Probst J, DiStefano C (2015). Factors affecting job satisfaction among agency-employed home health aides. Home Health Care Manag Pract.

[54] Lee AA, Jang Y (2016). What makes home health workers think about leaving their job? The role of physical injury and organizational support. Home Health Care Serv Q.

[55.••] Bien E, Davis K, Gillespie G (2020). Home healthcare workers' occupational exposures. Home Healthc Now.

[56] Gershon RR, Dailey M, Magda LA, Riley HE, Conolly J, Silver A (2012). Safety in the home healthcare sector: development of a new household safety checklist. J Patient Saf.

[57.••] Angus K, Semple S (2019). Home health and community care workers' occupational exposure to secondhand smoke: a rapid literature review. Nicotine Tob Res.

[58.••] Howard N, Adams D (2019). Home care services: an examination of the Washington state workers’ compensation claims data, 2012-2016. Technical Report Number 95-02-2019.

[59] Clyde M, Phillips L. Caregiver kicks (presentation slides). Service Employees International Union (SEIU) 775 Benefits Group; 2019. https://www.cdc.gov/nora/councils/hcsa/pdfs/14-ClydePhillips-508.pdf. Accessed 30 Mar 2021.

[60] Love M, Tendick-Matesanz F, Thomason J, Carter D, Glassman M, Zanoni J (2017). "Then they trust you …": managing ergonomics in home care. New Solut.

[61] Johnson S, McLeod K, Engel P, Tulloch L (2016). Musculoskeletal disorders and health profile of continuing care aides:an urban-rural comparison. Home Health Care Manag Pract.

[62] Hittle B, Agbonifo N, Suarez R, Davis KG, Ballard T (2016). Complexity of occupational exposures for home health-care workers: nurses vs. home health aides. J Nurs Manag.

[63] Faucett J, Kang T, Newcomer R (2013). Personal service assistance: musculoskeletal disorders and injuries in consumer-directed home care. Am J Ind Med.

[64] Sun C, Buchholz B, Quinn M, Punnett L, Galligan C, Gore R (2018). Ergonomic evaluation of slide boards used by home care aides to assist client transfers. Ergonomics..

[65] National Institute for Occupational Safety and Health. Safe Patient Handling and Mobility (SPHM). U.S. Department of Health and Human Services, Centers for Disease Control and Prevention; 2013. https://www.cdc.gov/niosh/topics/safepatient/default.html. Accessed 30 Mar 2021.

[66] Waters T (2007). When is it safe to manually lift a patient?. Am J Nurs.

[67] Beauvais A, Frost L (2014). Saving our backs: safe patient handling and mobility for home care. Home Healthc Nurse.

[68.••] Karlsson ND, Markkanen PK, Kriebel D, Gore RJ, Galligan CJ, Sama SR (2019). Home care aides’ experiences of verbal abuse: a survey of characteristics and risk factors. Occup Environ Med.

[69] Karlsson ND, Markkanen PK, Kriebel D, Galligan CJ, Quinn MM (2020). “That's not my job”: a mixed methods study of challenging client behaviors, boundaries, and home care aide occupational safety and health. Am J Ind Med.

[70] SEIU775 Benefits Group. HADit. 2020. https://seiu775.org/hadit/. Accessed 30 Mar 2021.

[71] Small TF, Gillespie GL, Kean EB, Hutton S. Workplace violence interventions used by home healthcare workers: an integrative review. Home Healthc Now. 2020;38(4):193–201. 10.1097/NHH.0000000000000874. 10.1097/NHH.0000000000000874 PMC12306958 32618777

[72] Ridenour ML, Hendricks S, Hartley D, Blando JD (2019). New Jersey home health care aides survey results. Home Health Care Manag Pract.

[73] Lipscomb JA, El Ghaziri M (2013). Workplace violence prevention: improving front-line health-care worker and patient safety. New Solut.

[74] Massachusetts Nurses Association. Workplace violence prevention materials booklet. 2008. http://www.massnurses.org/files/file/Health-and-Safety/Workplace-Violence/Workplace_Violence_booklet.pdf. Accessed 30 Mar 2021.

[75] McPhaul K, London M, Murrett K, Flannery K, Rosen J, Lipscomb J (2008). Environmental evaluation for workplace violence in healthcare and social services. J Saf Res.

[76] U.S. Department of Labor, Occupational Safety and Health Administration. Guidelines for preventing workplace violence for healthcare and social service workers. OSHA 3148-04R. 2015. https://www.osha.gov/Publications/osha3148.pdf. Accessed 30 Mar 2021.

[77] U.S. Government Accountability Office. Additional efforts needed to help protect health care workers from workplace violence. GAO-16-11. Mar 17, 2016. https://www.gao.gov/products/GAO-16-11. Accessed 30 Mar 2021.

[78.••] Brouillette NM, Quinn MM, Kriebel D. Risk of sharps injuries to home care nurses and aides: a systematic review and meta-analysis. J Occup Environ Med. 2017. 10.1097/JOM.0000000000001160. **Synthesizes data from a set of large U.S. NIOSH-funded studies of sharps injuries among home care nurses and aides.** 10.1097/JOM.0000000000001160 PMC5671783 28930800

[79] Brouillette NM, Quinn MM, Kriebel D, Markkanen PK, Galligan CJ, Sama SR, Gore RJ, Laramie AK, Davis L (2017). Risk of sharps injuries among home care aides: results of the Safe Home Care survey. Am J Infect Control.

[80] Goodyear N, Markkanen P, Beato-Melendez C, Mohamed H, Gore R, Galligan C, Sama S, Quinn M (2018). Cleaning and disinfection in home care: a comparison of 2 commercial products with potentially different consequences for respiratory health. Am J Infect Control.

[81] Agbonifo N, Hittle B, Suarez R, Davis K (2017). Occupational exposures of home healthcare workers. Home Healthc Now.

[82] Quinn MM, Henneberger PK (2015). Working Group on Cleaning and Disinfecting in Healthcare. Cleaning and disinfecting environmental surfaces in health care: toward an integrated framework for infection and occupational illness prevention. Am J Infect Control.

[83] Reckrey JM (2020). COVID-19 confirms it: paid caregivers are essential members of the healthcare team. J Am Geriatr Soc.

[84] Bandini J, Rollison J, Feistel K, Whitaker L, Bialas A, Etchegaray J (2021). Home care aide safety concerns and job challenges during the COVID-19 pandemic. New Solut.

[85] Madewell ZJ, Yang Y, Longini IM, Halloran ME, Dean NE (2020). Household transmission of SARS-CoV-2: a systematic review and meta-analysis. JAMA Netw Open.

[86] Munoz-Price LS, Rivera F, Ledeboer N. Air contamination of households versus hospital inpatient rooms occupied by SARS-CoV-2 positive patients. Infect Control Hosp Epidemiol. 2021:1–14. 10.1017/ice.2021.45. 10.1017/ice.2021.45 PMC8047394 33536089

[87] Caban-Martinez AJ, Arlinghaus A, Reme SE (2013). Correlates of seasonal flu vaccination among U.S. home health aides. Vaccine..

[88.••] Muramatsu N, Sokas RK, Chakraborty A, Zanoni JP, Lipscomb J (2018). Slips, trips, and falls among home care aides: a mixed-methods study. J Occup Environ Med.

[89] Betsy Lehman Center for Patient Safety. From the frontlines: home care workers voice COVID-19 challenges and ideas for change. January 2021. https://betsylehmancenterma.gov/assets/uploads/FromTheFrontlines_Report.pdf. Accessed 30 Mar 2021.

[90] Bercovitz A, Moss A, Sengupta M, Park-Lee EY, Jones A, Harris-Kojetin LD. An overview of home health aides: United States, 2007. Natl Health Stat Rep. 2011;(34):1–31. 21688727

[91] Poo A-j. The pandemic offers a chance to reimagine caregiving: to build back better, Biden’s administration should focus on one of this country’s fastest-growing occupations: home care work. The Nation, 2021. https://www.thenation.com/article/society/pandemic-caregiver-biden/. Accessed 30 Mar 2021.

[92] Campbell J, Koca F (2021). Financing and protection for the health and care workforce. Bull World Health Organ.

